# Modulation of host inflammatory pathways by *Pseudomonas aeruginosa* extracellular vesicles in cystic fibrosis: impact of pulmonary exacerbation and elexacaftor-tezacaftor-ivacaftor treatment

**DOI:** 10.3389/fimmu.2026.1745853

**Published:** 2026-01-22

**Authors:** Marianna Said, Aszia Burrell, Brennan Harmon, Kylie I. Krohmaly, Marina Mazur, George M. Solomon, Andrea Hahn

**Affiliations:** 1 Center for Precision Medicine and Genomics Research, Children’s National Research Institute, Washington, DC, United States; 2 Sheikh Zayed Institute for Pediatric Surgical Innovation, Children’s National Research Institute, Washington, DC, United States; 3 Department of Medicine, University of Alabama at Birmingham, Birmingham, AL, United States; 4 Gregory Fleming James Cystic Fibrosis Research Center, University of Alabama at Birmingham, Birmingham, AL, United States; 5 Division of Infectious Diseases, Children’s National Hospital, Washington, DC, United States; 6 Department of Pediatrics, George Washington University School of Medicine and Health Sciences, Washington, DC, United States

**Keywords:** epithelial cells, cystic fibrosis, cytokines, extracellular vesicles, *Pseudomonas aeruginosa*

## Abstract

**Background:**

*Pseudomonas aeruginosa* is a prevalent pathogen in persons with cystic fibrosis (pwCF), frequently causing chronic infections. These infections are often associated with episodic pulmonary exacerbations (PEx), yet the mechanisms linking changes in *P. aeruginosa* to these PEx remain poorly understood. It is unknown whether virulence changes in *P. aeruginosa* extracellular vesicles (EVs) contribute to PEx. We hypothesized that *P. aeruginosa* EVs from PEx will elicit increased inflammation response compared to Pa EVs from clinical stability in an *in vitro* cell culture model.

**Methods:**

*P. aeruginosa* EVs were isolated from the sputum of pwCF collected at PEx and the immediately preceding clinical stability period. *P. aeruginosa* EVs or MEM control were applied apically to primary human bronchial epithelial (HBE) cells at air-liquid interface in the presence or absence of elexacaftor/tezacaftor/ivacaftor (ETI). Basal media and apical cell secretions were measured for 10 inflammatory cytokines using MesoScale Discovery (MSD) assays. RNAseq was performed on the HBE cells, with differential canonical pathways identified using Ingenuity Pathway Analysis (IPA).

**Results:**

*P. aeruginosa* EVs at PEx induced reduced cytokine production in both HBE donors in the presence of ETI (p<0.001 except apical TNF-α p=0.022). The IPA results, which were hypothesis generating, suggested that the S-100 family pathway may be another measure of inflammation that is useful to study in CF.

**Conclusions:**

*P. aeruginosa* EVs induced HBE cell inflammation, which was reduced in the presence of ETI.

## Introduction

Cystic fibrosis (CF) is a life-shortening autosomal recessive disease caused by mutations in the cystic fibrosis transmembrane conductance regulator (CFTR) gene and affects over 105,000 people worldwide (1). CFTR encodes an anion channel responsible for chloride and bicarbonate transport across epithelial surfaces. Loss of functional CFTR impairs ion and water transport, resulting in dehydrated, viscous airway mucus that disrupts mucociliary clearance (2). Altered epithelial cell signaling leads to recruitment of neutrophils and constitutively activated nuclear factor kappa B (NF-κB) (3). This altered airway environment promotes chronic infections, persistent inflammation, and activation of pro-inflammatory cytokines (e.g., interleukin (IL)-6 and IL-8) (4, 5). Recurrent acute episodes of lung infection and inflammation, termed pulmonary exacerbations (PEx), accelerate the decline in lung function (6). Over time, this cycle of infection and inflammation drives progressive lung function decline, respiratory failure, and mortality (7).

*Pseudomonas aeruginosa* is among the most common pathogens in cystic fibrosis with a current annual prevalence of approximately 25% (8). Historically *P. aeruginosa* was more frequently reported, with a lifetime occurrence in up to 90% of patients (8, 9). The recent reduction in *P. aeruginosa* infection rates is likely driven by the increased use of highly effective CFTR modulators (10, 11). However, this remains an important pathogen for study as chronic infections with *P. aeruginosa* persist and are known to drive lung inflammation in CF (12, 13). Notably, changes in relative abundance or bacterial load are not sufficient to account for the onset of PEx (14). Studies suggest that *P. aeruginosa* extracellular vesicles (EVs) carry virulence factors capable of modulating host immune response, thereby contributing to inflammation and disease progression (15). However, it is unknown whether these virulence factors specifically alter lung inflammation and contribute to pulmonary exacerbations.

We hypothesized that *P. aeruginosa* EVs are contributing to pulmonary exacerbations by inducing an inflammatory response in airway epithelial cells. Furthermore, we hypothesized that elexacaftor-tezacaftor-ivacaftor (ETI) would dampen the inflammatory response, but that differences would still be present between pulmonary exacerbation and clinical stability. To test our hypotheses, we measured inflammatory cytokines and changes in the transcriptome of primary human bronchial epithelial cells exposed to *P. aeruginosa* EVs – either from PEx or clinical stability – in the presence and absence of ETI.

## Materials & methods

### Study design

The experiments in this study were designed to test the hypothesis that *P. aeruginosa* EVs are contributing to pulmonary exacerbations. Eight paired sputum samples from persons with CF who were culture positive for *P. aeruginosa* were selected from our biorepository. The first sample selected was from a time the person with CF was experiencing a pulmonary exacerbation. The second sample was the immediately preceding sample from a time when the person with CF was at their baseline (e.g., clinical stability). EVs were isolated from the supernatants, as described below. We then applied the EVs to primary human bronchial epithelial cells at air-liquid interface (ALI) from two different donors, with and without treatment with ETI, as described below. Apical secretions, cells, and basal media were all harvested, and flash frozen in dry ice prior to freezing at -80°C until testing was performed.

The biorepository from which the sputum samples were selected is IRB approved (Children’s National IRB Pro6781, original approval 8 December 2015 with yearly continuing reviews). Consent is obtained for participation in the biorepository from adults; for children < 18 years of age, parental permission is obtained with assent from children 11–17 years of age. The experiments using the de-identified donor cells were also IRB approved (Children’s National IRB Pro13142, approved 24 March 2020, with continuing reviews not required).

### Respiratory sample collection and processing

Respiratory samples were collected, processed, and stored as previously described (16, 17). Briefly, sputum samples are collected in sterile containers and stored at 4°C until processing. Sputum is mixed 1:1 volume/volume with sterile saline and Sputasol (Thermofisher), vortexed, and heated at 37°C for 10 minutes. The homogenized sputum is then aliquoted and centrifuged (12000*g* x 10 minutes). Supernatants are removed, and are stored separately from cell pellets at -80°C for long-term storage.

### Isolation of *Pseudomonas aeruginosa* EVs from sputum samples

*P. aeruginosa* EVs were isolated as previously described (18). Briefly, 1 mL frozen sputum supernatants were thawed and mixed with ExoQuickTC (Systems Biosciences) for precipitation, following the manufacturer’s instructions. The isolated EVs were further purified via size exclusion chromatography using qEV (Izon Science). The 1^st^-3^rd^ collections from the qEV isolation were combined and mixed with ExoQuick (Systems Biosciences) to concentrate the EVs. This was followed by resuspension of the EVs in 1 mL of phosphate buffered solution (PBS). Next, *P. aeruginosa*-specific OMP antibodies (mouse monoclonal isotype IgG2a, Abcam) were used to isolate *P. aeruginosa*-specific EVs (19).

### Nanoparticle tracking of *Pseudomonas aeruginosa* EVs

We used Spectradyne nCS1™ to measure the size and concentration of *P. aeruginosa* EVs as previously described (18). Briefly, 5 uL of 1:1 diluted sample (0.02 micron-filtered PBS with 1% TWEEN) were analyzed on C-400 cartridges (Spectradyne LLC). Default voltages and settings were used, until an N of ~400 particles were analyzed (5% error). Peak filters were applied to limit the particle diameters between 70 nm and 400 nm, and then EV concentration and diameter were calculated. *P. aeruginosa* EVs were diluted using minimum essential medium (MEM) without phenol red to normalize the EV concentration across samples so that the same EV exposure would occur in subsequent cell culture experiments.

### Validation of *Pseudomonas aeruginosa* EV specificity

To further characterize and validate that our isolation techniques were sufficient to isolate *P. aeruginosa*-specific EVs, we conducted proteomics of a subset of the isolated EVs, which has been published elsewhere (18). Briefly, we used patient-derived EVs, which we called *P. aeruginosa* EVs (sputum) and control *P. aeruginosa* EVs isolated from pure culture, which we called *P. aeruginosa* EVs (culture). Protein peptides were measured using a mass spectrometer integrated with a UPLC system, and mass spectra were acquired using data-independent acquisition mode. We conducted proteomic data analysis using Spectronaut™, using both the reviewed only (i.e., UniProtKB/Swiss-Prot) and reviewed and unreviewed (i.e., UniProtKB TrEMBL) *P. aeruginosa* protein databases. We also ran our peptide output against a human proteome database to assess purity of our isolated EVs.

### Exposure of primary human bronchial epithelial cells to *Pseudomonas aeruginosa* EVs

Primary human bronchial epithelial cells (dF508 homozygous) from two CF donors were grown for 4 weeks at 37°C to air-liquid interface in Pneumacult^®^ differentiation media. After differentiation, half of the cells were pretreated with elexacaftor/tezacaftor/ivacaftor (ETI; doses of 3 µM, 5 µM, and 0.03 µM, respectively) in the basal compartment for 48 hours, re-dosed at 24 hours. After incubation, the basal media was changed – with the addition of a third dose of ETI – and the apical secretions were washed with 100 uL PBS before exposing the cells to equal volumes (50 µL) of *P. aeruginosa* EVs (in duplicate) or with minimum essential medium (MEM) as a negative control. Following another 24-hour incubation, 200 uL of PBS was added to the apical layer and allowed to sit at 37°C for 30 minutes. Next, 200 uL apical secretions and 500 uL basal media were removed from the cell culture and flash frozen using dry ice. Likewise, cells from 70 filters were harvested using ice-cold TRIzol and flash frozen using dry ice. All apical secretions, basal media, and HBE cells were frozen at -80°C until MesoScale Discovery (MSD) analysis or RNA extraction, respectively.

### Meso Scale Discovery assays of apical and basal media

Inflammatory cytokines were quantified from both apical secretions and basal media using the MSD V-PLEX Proinflammatory Panel 1 (human) on the MESO QuickPlex SQ 120 system. The panel included interleukin (IL)-1β, IL-2, IL-4, IL-6, IL-8, IL-10, IL-12p70, IL-13, tumor necrosis factor (TNF)-α, and interferon (IFN)-γ. 25 µL of each sample was loaded per well, and all samples were run in duplicate. For IL-8, concentrations in undiluted samples exceeded the assay’s upper detection limit, but serial dilutions determined that a 1:100 dilution fell within the standard curve. Due to limited sample availability, IL-8 was assessed only in apical secretions and not in basal media. Plate washing, incubation, and detection steps were performed according to the manufacturer’s instructions.

### RNA extraction and sequencing of epithelial cells

Total RNA was isolated from cells using Trizol (ThermoFisher Scientific, Waltham, MA) in combination with the Direct-zol RNA Microprep Kit (Zymo Research, Irvine, CA). RNA quantity was measured using the Qubit™ RNA Broad-Range Assay Kit (ThermoFisher Scientific, Waltham, MA) and RNA quality was assessed with the Agilent TapeStation (Agilent, Palo Alto, CA). Total RNA was prepared for sequencing using the NEBNext Ultra II Directional RNA Library Prep Kit (New England Biolabs, Ipswich, MA) using a polyA enrichment approach. The resultant sequencing libraries were sequenced on an Illumina NovaSeq6000 sequencer using a S4 100PE Flowcell (Illumina, San Diego, CA).

### Statistical and bioinformatic analysis

To account for inter-plate variability across multi-plate MSD experiments, cytokine concentrations were normalized prior to statistical analysis. Each plate was run with its own set of standards, and for each analyte, all standards were plotted together to assess linearity and variability. The optimal standard for normalization was selected based on low intra- and inter-plate variation, strong linearity, and high concentration. Standard 2 was chosen for all analytes except IL-12p70 and TNF-α, for which standard 3 was used. For each analyte, the median calculated concentration of the chosen standard across plates was determined. A plate-specific normalization factor was then generated by dividing this median value by the calculated concentration of the chosen standard for that plate. The average concentration for each well was multiplied by the plate-specific normalization factor to yield normalized cytokine concentrations for each analyte, which were used for subsequent statistical analyses. Definitions and formulas to summarize these calculations are as follows:

- Calculated Concentration Mean = the mean concentration of each sample or standard run in duplicate.
- Plate-Specific Normalization Factor = the median calculated concentration mean across all plates/the calculated concentration mean for each standard on each plate.
- Normalized Concentration Mean = Calculated Concentration Mean * Plate-Specific Normalization Factor.

STATA/IC 15.1 was used for statistical analyses of MSD data. As the MSD assays measured cytokines from two primary epithelial cell donors, and also included paired exposure samples in some comparisons (pulmonary exacerbation versus stable), a GLS random-effects linear model was used to determine significance between exposure variables (xtreg, re), selecting donor as the panel variable (xtset). As the MSD assays tested for 10 cytokines each in the apical media and basal secretions, and the experiment included 2 donor cells (n=40 comparisons), a Bonferroni correction was used with p<0.00125 considered significant.

Differential gene expression of the RNAseq data was determined using a Galaxy workflow incorporating HISAT2, featureCounts, and DESeq2. The resultant gene expression table, including the expression log ratio, p-value, and false-discovery rate, was taken into Ingenuity Pathway Analysis (IPA, Qiagen). Biological filters used in the core analysis of these datasets within IPA included human as species, and lung as the tissue and cell line. A log2 fold change ≥|0.5| of the expression log ratio and a p-value of 0.05 was set as a cutoff for incorporating genes in the IPA analysis. Both direct and indirect relationships were considered in predicting activated canonical pathways and upstream regulators. Z-scores ≥|2| were considered significant.

## Results

### Demographics of cell and sputum donors for *in vitro* experiments

Two people with CF donated the primary cells that were used in our cell culture experiments. Donor 1 was an 18-year-old White female, and Donor 2 was a 28-year-old White male. Both donors had a CFTR genotype that was homozygous for the F508del mutation. Paired sputum samples obtained from 8 people with CF who had sputum cultures positive for growth of *P. aeruginosa* were used to isolate the *P. aeruginosa* EVs. The mean age of the sputum donors was 21 years (SD 6.1, range 10–31 years). The mean number of days between the sputum samples obtained at clinical stability and at subsequent pulmonary exacerbation was 67 days (SD 21.3, range 28–91 days). The mean percent predicted forced expiratory volume in one second (ppFEV_1_) at the time of clinical stability was 88% (SD 14.4%, range 70-109%), whereas the mean ppFEV_1_ at the time of pulmonary exacerbation was 82% (SD 12.6%, range 64-99%).

### *Pseudomonas aeruginosa* EV characteristics for *in vitro* experiments

The mean diameter of the 90^th^ percentile of the isolated *P. aeruginosa* EVs was 165 nm (SD 26.5 nm). The mean measured concentration was 1.72E9 particles/mL (SD 3.14E9 particles/mL). The *P. aeruginosa* EVs were then diluted with MEM at various ratios to normalize the concentrations across samples so that the EV exposure would be similar across experiments. This resulted in a mean concentration of 9.36 million particles in 50 µL (SD 2 million) such that the calculated ratio of *P. aeruginosa* EVs/cell was 37.4 (SD 8). Evaluating the proteomics results from the *P. aeruginosa* reviewed only protein database (i.e., UniProtKB/Swiss-Prot), a total of 983 P*. aeruginosa* proteins were identified; 982 proteins were detected in the control, whereas a mean of 7.4 proteins per sample were detected in 10 patient-derived EVs. Notably, we did not detect a significant number of human proteins using our EV isolation techniques. Additional details about these validation experiments have been published elsewhere (18).

### Meso Scale Discovery inflammatory cytokine panel

**Figure 1:**
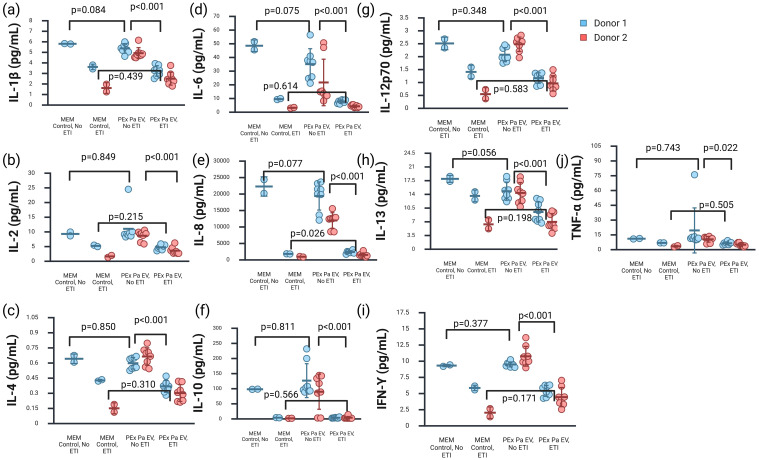
Inflammatory cytokines were increased in the apical secretions of primary cystic fibrosis cells exposed to *Pseudomonas aeruginosa* extracellular vesicles in the absence of elexacaftor-tezacaftor-ivacaftor (ETI) treatment compared to treated cells. Error bars represent the standard deviation surrounding the mean. P-values shown are for the GLS random-effects linear model for the dependent variable ETI exposure, and setting donor as the panel variable. **(a)** Interleukin (IL)-1β. **(b)** IL-2. **(c)** IL-4. **(d)** IL-6. **(e)** IL-8. **(f)** IL-10. **(g)** IL-12p70. **(h)** IL-13. **(i)** Interferon (IFN)-γ. **(j)** Tumor necrosis factor (TNF)-α. Created in BioRender. Hahn, A. (2026) https://BioRender.com/q143xqt.

**Figure 2:**
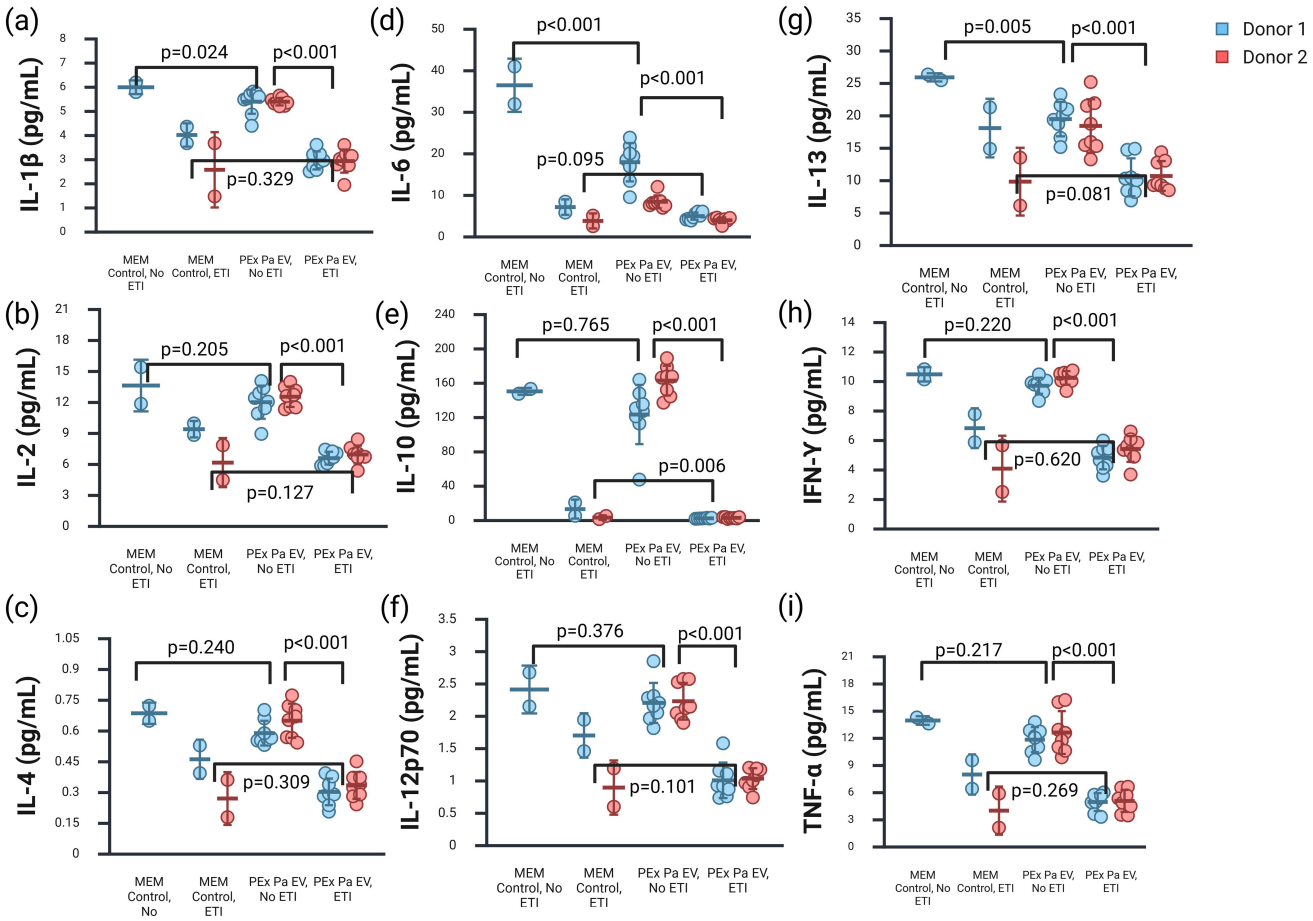
Inflammatory cytokines were increased in the basal media of primary cystic fibrosis cells exposed to *Pseudomonas aeruginosa* extracellular vesicles in the absence of elexacaftor-tezacaftor-ivacaftor (ETI) treatment compared to treated cells. Error bars represent the standard deviation surrounding the mean. P-values shown are for the GLS random-effects linear model for the dependent variable ETI exposure, and setting donor as the panel variable. **(a)** Interleukin (IL)-1β. **(b)** IL-2. **(c)** IL-4. **(d)** IL-6. **(e)** IL-10. **(f)** IL-12p70. **(g)** IL-13. **(h)** Interferon (IFN)-γ. **(i)** Tumor necrosis factor (TNF)-α. IL-8 results were above the upper limit of quantification for the assay (data not shown). Created in BioRender. Hahn, A. (2026) https://BioRender.com/pwhdljf.

When comparing the cytokine measurements between cells exposed to *P. aeruginosa* EVs isolated from sputum obtained during pulmonary exacerbation to cells exposed to MEM controls, we did not see any significant differences in either the apical secretions (**Figure 1**; **Supplementary Table 1**) or basal media (**Figure 2**; **Supplementary Table 2**) with the exception of IL-6 being significantly increased in the control compared to the EVs (Donor 1 Pa EVs 17.8 pg/mL and Donor 2 Pa EVs 8.3 pg/mL vs Donor 1 control 36.3 pg/mL, p<0.001). This finding was unexpected, but may be related in part to the absence of a control sample for Donor 2. There were also minimal differences based on cell donor, except for IL-8 in the apical secretions and IL-6 in the basal media.

With the addition of ETI treatment to our experimental model, we did not see any significant differences between cells exposed to *P. aeruginosa* EVs isolated from sputum obtained during pulmonary exacerbation to cells exposed to MEM controls. When evaluating the impact of cell donor, differences were noted in the apical secretion of IL-1β, IL-2, IL-6, and IL-8, but these differences were not statistically assessed.

Similarly, we did not see any significant differences when comparing cytokine measurements between cells exposed to *P. aeruginosa* EVs isolated from sputum obtained during pulmonary exacerbation to cells exposed to *P. aeruginosa* EVs isolated from sputum obtained during clinical stability in either the apical secretions (**Supplementary Table 1**; **Supplementary Figures 1, 2**) and basal media (**Supplementary Table 2**; **Supplementary Figures 3, 4**). This was true both in the presence and absence of treatment with ETI. However, differences in cytokine measurements were again noted based on donor cells, specifically for IL-6 and IL-8 in the apical secretions without ETI, IL-1β, IL-2, and IL-6 in the apical secretions with ETI, IL-6 and IL-10 in the basal media without ETI, and IL-6 in the basal media with ETI.

We did see significant differences in the majority of our 10 cytokine measurements from cells exposed to *P. aeruginosa* EVs isolated from sputum during pulmonary exacerbation in both the apical secretions and basal media, based on whether the cells were treated with ETI during the experiments (**Figures 1**, **2**). We also noted differences between cell donors for some specific cytokines measured, namely IL-1β and IL-8 in the apical secretions and IL-6 in the basal media.

### Transcriptomics and Ingenuity Pathway Analysis

RNA quality assessment demonstrated high RNA integrity numbers (mean 9.986, SD 0.20). Differentially abundant transcripts were taken into IPA as a hypothesis-generating method to identify predicted changes in canonical pathways and upstream regulators (**Supplementary Figure 5**). We evaluated the canonical pathways associated with the cytokines tested in our MSD assays, as well as other significant inflammatory canonical pathways and upstream regulators (**Table 1**). Similar to our MSD results, there were not many significant differences noted when comparing *P. aeruginosa* EVs from pulmonary exacerbation to MEM controls or clinical stability, with a few exceptions. In donor 2 cells treated with ETI, IL-1β, IL-4, chemokine CXC motif ligand 8 (CXCL8; i.e., IL-8) and TNF were predicted to be significantly increased upstream regulators in the cells exposed to *P. aeruginosa* EVs from pulmonary exacerbation versus MEM controls.

**Table 1:** Select inflammatory canonical pathways and upstream regulators predicted by ingenuity pathway analysis.

Pathway or upstream regulator	Z score, Pa PEx EVs vs MEM controls, no ETI, donor 1	Z score, Pa PEx EVs vs MEM controls, with ETI, donor 1	Z score, Pa PEx EVs vs MEM controls, with ETI, donor 2	Z score, Pa EVs clinical stability vs PEx, no ETI, donor 1	Z score, Pa EVs clinical stability vs PEx, no ETI, donor 2	Z score, Pa EVs clinical stability vs PEx, with ETI, donor 1	Z score, Pa EVs clinical stability vs PEx, with ETI, donor 2	Z score, Pa PEx EVs no ETI vs with ETI, donor 1	Z score, Pa PEx EVs no ETI vs with ETI, donor 2
Select inflammatory canonical pathways									
S100 Family Signaling Pathway	1.134	-0.535	0.272	NaN	-0.707	-1.633	-2.449*	4.352*	6.235*
Role of Pattern Recognition Receptors in Recognition of Bacteria and Viruses	NR	NaN	0	NR	NaN	NR	NaN	0	1.877
Antimicrobial peptides	NR	NR	NaN	NR	NR	NR	NR	1.5	3.371*
Neutrophil degranulation	0	0	1.461	NaN	NaN	0	NaN	1.016	1.298
Interleukin-1 family signaling	NR	NaN	NaN	NaN	NaN	NaN	NR	-3.727*	1.3
IL-1 signaling	NR	NR	NaN	NR	NaN	NaN	NaN	-2.324*	1.291
Interleukin-2 family signaling	NR	NaN	-0.447	NR	NR	NR	NaN	-0.5	0.258
IL-2 signaling	NR	NaN	-1.342	NR	NR	NaN	NaN	-0.943	1.604
IL-4 Signaling	NaN	0.447	0.535	NaN	NaN	NaN	NR	2.183*	3.781*
Interleukin-6 family signaling	NaN	NaN	NaN	NaN	NR	NR	NR	-0.632	-0.632
IL-6 signaling	NR	NaN	0	NR	NR	NaN	NR	-1.897	2.263*
IL-8 signaling	NaN	-0.447	0	NaN	NaN	NaN	NaN	1.344	4.389*
Interleukin-10 signaling	NR	NR	NR	NaN	NaN	NaN	NaN	-0.408	1.147
IL-10 signaling	NaN	NaN	-1.508	NR	NaN	NaN	NR	1.443	0.309
Interleukin-12 family signaling	NR	NaN	NaN	NR		NR	NaN	0.905	0
IL-12 signaling and production in macrophages	NR	1.342	-0.775	NaN	NaN	NaN	NaN	1.344	1.313
IL-13 signaling pathway	NaN	NaN	0.832	NR	NaN	NR	NR	0.522	2.121*
Interleukin-4 and Interleukin-13 signaling	NR	-0.816	0.258	NR	NaN	NR	NR	0.775	2.06*
Interferon signaling	NR	NR	NaN	NaN	NR	NR	NR	-2.309*	-1.414
Interferon gamma signaling	NR	NaN	0.378	NR	NaN	NaN	NR	-2.466*	-1.219
TNF signaling	NR	NR	NR	NR	NR	NR	NR	-1.606	0.302
Select inflammatory upstream regulators									
TGFB1	NR	NR	0.044	NR	NaN	NR	NR	-0.297	2.469*
IL1B	NR	NaN	2.236*	NR	NR	NR	NR	NR	1.998
CCL2	NR	NaN	2*	NR	NR	NR	NR	2.111*	1.265
TSLP	NR	NaN	2*	NR	NR	NR	NR	2.111*	1.265
CXCL8	NR	NaN	2*	NR	NR	NR	NR	2.111*	1.265
IL33	NR	NaN	2*	NR	NR	NR	NR	1.732	1.265
IL4	NR	NaN	2*	NR	NR	NR	NR	1.732	1.732
TNF	NR	NaN	2*	NR	NaN	NR	NR	1.233	1.747

*Z score ≥ |2| is considered significant. Positive Z-scores are increased in the first comparator, whereas negative Z-scores are increased in the second comparator. IL, interleukin; TNF, tumor necrosis factor; TFGB1, transforming growth factor beta 1; CCL2, CC motif chemokine ligand 2; TSLP, thymic stromal lymphopoietin; CXCL8, chemokine CXC motif ligand 8; NaN, not a number; NR, not reported.

When comparing cells treated without or with ETI following exposure to *P. aeruginosa* EVs isolated from pulmonary exacerbation, we saw both significant decreases (donor 1 only) and increases (donor 1 and 2) in pathways associated with the previously tested cytokines (**Table 1**). Cytokines that were noted to be significantly increased included IL-4, IL-6, IL-8, and IL-13. Cytokines noted to be significantly decreased included IL-1 and IFN.

Interestingly, other inflammatory pathways that were not tested in our MSD assay seemed to more closely align with our hypothesis that *P. aeruginosa* EVs are able to induce inflammation. The S100 family signaling pathway was noted to be significantly decreased in donor 2 cells exposed to *P. aeruginosa* EVs isolated from clinical stability compared to those isolated at pulmonary exacerbation (Z-score -2.449). Furthermore, the S100 family signaling pathway was significantly increased in cells exposed to *P. aeruginosa* EVs isolated from pulmonary exacerbation without compared to with ETI treatment (donor 1 Z-score 4.352, donor 2 Z-score 6.235). Other infection and inflammatory pathways that were significantly increased or approached significance in cells following *P. aeruginosa* EV exposure include the role of pattern recognition receptors in recognition of bacteria and viruses, antimicrobial peptides, and neutrophil degranulation (**Table 1**). A full list of all reported canonical pathways in our dataset is available in the supplement (IPA Canonical Pathways_supplement).

We also evaluated additional upstream regulators predicted to be activated based on the genes up- or down-regulated in our dataset. Regulators that were predicted to be activated following exposure to *P. aeruginosa* EVs isolated at pulmonary exacerbation across multiple comparisons included transforming growth factor beta 1 (TGFB1), CC motif chemokine ligand 2 (CCL2), and thymic stromal lymphopoietin (TSLP) (**Table 1**). A full list of all reported upstream regulators in our dataset is available in the supplement (IPA Upstream Regulators_supplement).

## Discussion

With this study, our research group again demonstrated that *P. aeruginosa* EVs can independently induce inflammation in CF human bronchial epithelial cells, this time using an ALI model in the presence and absence of ETI treatment. As expected, ETI diminished the inflammatory response. Contrary to our hypothesis, we did not see any consistent differences based on the clinical state of the person with CF from whom the *P. aeruginosa* EVs were isolated. These findings may suggest that *P. aeruginosa* EV content is similar across timepoints, or they may suggest that other clinical factors are more important in driving the symptoms of pulmonary exacerbation. Unfortunately, these questions cannot be answered by our experimental design and should be considered in future experiments.

Based on prior studies, we anticipated that IL-1β, IL-6 and IL-8 would be important inflammatory cytokines in our model (5, 20). We did not see differences in either direct cytokine measurement of the apical secretions or basal media when comparing exposure of *P. aeruginosa* PEx EVs to either MEM control or *P. aeruginosa* EVs from clinical stability. Interestingly, all three of these cytokines demonstrated significant differences in levels in the apical secretions based on the cell donor, demonstrating individual differences in response despite having the same genotype (dF508). When evaluating canonical pathways based on cell transcriptomic data, we did see a significant increase in the upstream regulators IL-1β, IL-4 CXCL-8 (i.e., IL-8), and TNF when exposed to *P. aeruginosa* PEx EVs compared to MEM control (for donor 2 only). These findings of *P. aeruginosa* EVs inducing an inflammatory response are consistent with our prior work (18), as well as the work of others who have shown specific increases in IL-1β, IL-6, and IL-8, among other cytokines, in cell culture and mouse models (21–23).

We did see significant differences in IL-1β, IL-6, and IL-8 (as well as the other 7 measured cytokines) when comparing *P. aeruginosa* EV exposure in the presence and absence of ETI. This is partially consistent with the clinical experience, where sputum levels of IL-1β and IL-8 were reduced after initiation of ETI, whereas sputum IL-6 levels were increased (24).

The strongest association we found in our study was with the S100 family signaling pathway based on our cell transcriptomic data. S100 family signaling was significantly increased with *P. aeruginosa* PEx EV exposure compared to MEM controls for donor 1 in the presence and absence of ETI, as well as with exposure to *P. aeruginosa* PEx EVs in absence of ETI compared to the presence of ETI for both donors 1 and 2. S100A8, S100A9, and S100A12 – also known as calgranulins A, B, and C respectively – are damage-associated molecular pattern (DAMP) molecules released by neutrophils as part of the inflammatory response (25). Notably, they induce mucin expression through the NF-κB pathway (25), suggesting they may also be important regulators of inflammation in persons with CF. A few studies have demonstrated that these S100 family proteins are more frequently detected in people with CF (26–28), with two suggesting they may be elevated specifically during PEx (26, 28). However, it is important to note that these predictions are hypothesis generating, and future studies will require validation through qPCR or protein assays.

There remain limitations to our study design. While we normalized the exposure of the *P. aeruginosa* EV concentration across experiments, we do not know the specific EV contents that were interacting with the HBE cells. Furthermore, the concentration of EVs that we used may not have been sufficient to elicit the expected response in the human lung. Additionally, we only measured cytokine response at 24 hours, but different cytokines may peak sooner and return to baseline by 24 hours. To fully understand the *P. aeruginosa* EV response, future experiments should include variable concentrations of EVs as well as measurements at multiple time points. While we incorporated biologic variability into our cell culture model purposefully (8 persons with CF with paired sputum used to isolate the *P. aeruginosa* EVs), there could be different proteins or nucleic acids within the EVs, thereby making the comparisons between PEx and clinical stability more difficult to interpret. We also purposefully incorporated biologic variability by using two cell donors. We did note differences in the response of the donors, both in the presence and absence of ETI. This suggests differential response to ETI treatment even with the same genetic mutation, which is consistent with the clinical experience. At least one study has demonstrated variability in clinical outcomes after initiation of ETI (29), but further investigation is needed to explain these differences (30).

In summary, our preliminary results suggest that *P. aeruginosa* EVs may be able to induce an inflammatory response in a CF HBE cell culture model, specifically IL-1β, IL-6, IL-8, and the S100 family signaling pathway. These findings are hypothesis generating and should be validated in future studies. While the impact of *P. aeruginosa* EVs on inflammation was less than we hypothesized, this may be influenced by the limitations in our experimental design noted above. We did find that the inflammatory response was diminished with ETI treatment. We did not see a difference in inflammatory response based on the timing of the *P. aeruginosa* EV isolation, but interpreting this finding may be limited as we did not quantify the *P. aeruginosa* EV contents prior to exposure.

## Data Availability

The datasets presented in this study can be found in online repositories. The names of the repository/repositories and accession number(s) can be found below: https://www.ncbi.nlm.nih.gov/, BioProject PRJNA1363117.
