# Supplementary material for: Modulation of host inflammatory pathways by Pseudomonas aeruginosa extracellular vesicles in cystic fibrosis: impact of pulmonary exacerbation and elexacaftor-tezacaftor-ivacaftor treatment
Source: Front Immunol. 2026 Jan 22;17:1745853. doi: 10.3389/fimmu.2026.1745853 (PMC12872476; doi:10.3389/fimmu.2026.1745853)
Supplement: Supplementary file 1 [file DataSheet1.docx]

**Modulation of host inflammatory pathways by *Pseudomonas aeruginosa* extracellular vesicles in cystic fibrosis: impact of pulmonary exacerbation and elexacaftor-tezacaftor-ivacaftor treatment**

Marianna Said BS, Aszia Burrell BS, Brennan Harmon MS, Kylie I. Krohmaly PhD, Marina Mazur MS, George M. Solomon MD and Andrea Hahn MD, MS

**Supplemental Results**

**Supplemental Table 1. Measures of apical secretion inflammatory cytokines following *Pseudomonas aeruginosa* extracellular vesicle exposure**

| **Cytokine**  **(mean, SD)** | **Pa Ev at PEx, Donor 1** | **MEM control, Donor 1** | **Pa Ev at PEx, Donor 2** | **MEM control, Donor 2** | **P-value** |
| --- | --- | --- | --- | --- | --- |
| IL-1β | 5.337±0.454 | 5.773 ± 0.028 | 4.889 ± 0.522 | NP | 0.084 |
| IL-2 | 10.913 ± 5.468 | 9.174 ± 0.929 | 8.564 ± 1.672 | NP | 0.849 |
| IL-4 | 0.594 ± 0.058 | 0.638 ± 0.047 | 0.661 ± 0.087 | NP | 0.850 |
| IL-6 | 34.812 ± 11.310 | 48.270 ± 4.275 | 21.436 ± 16.993 | NP | 0.075 |
| IL-8 | 19288.64 ± 4279.183 | 22178.31 ± 2938.350 | 11852.460 ± 2348.669 | NP | 0.077 |
| IL-10 | 125.428 ± 56.076 | 96.911 ± 0.538 | 88.590 ± 58.004 | NP | 0.811 |
| IL-12p70 | 2.060 ± 0.272 | 2.497 ± 0.243 | 2.464 ± 0.279 | NP | 0.348 |
| IL-13 | 14.707 ± 2.311 | 17.859 ± 0.891 | 14.270 ± 2.655 | NP | 0.056 |
| IFN-ϒ | 9.438 ± 0.386 | 9.259 ± 0.159 | 10.699 ± 1.531 | NP | 0.377 |
| TNF-α | 19.118 ± 22.843 | 10.711 ± 0.348 | 10.114 ± 2.917 | NP | 0.743 |
|  | **Pa Ev at PEx with ETI, Donor 1** | **MEM control with ETI, Donor 1** | **Pa Ev at PEx with ETI, Donor 2** | **MEM control with ETI, Donor 2** | **P-value** |
| IL-1β | 3.202 ±0.494 | 3.577 ± 0.258 | 2.463 ± 0.611 | 1.577 ± 0.510 | 0.439 |
| IL-2 | 4.645 ± 0.789 | 5.10 ± 0.347 | 3.490 ± 1.222 | 1.528 ± 0.467 | 0.215 |
| IL-4 | 0.364 ± 0.059 | 0.424 ± 0.009 | 0.298 ± 0.083 | 0.147 ± 0.054 | 0.310 |
| IL-6 | 7.523 ± 1.193 | 9.278 ± 0.499 | 4.031 ± 0.871 | 2.922 ± 0.368 | 0.614 |
| IL-8 | 2285.845 ± 412.168 | 1706.573 ± 40.564 | 1403.75 ± 578.273 | 844.062 ± 135.713 | 0.026 |
| IL-10 | 2.438 ± 1.339 | 1.661 ± 0.418 | 2.689 ± 4.093 | 0.623 ± 0.309 | 0.566 |
| IL-12p70 | 1.155 ± 0.203 | 1.395 ± 0.260 | 0.950 ± 0.298 | 0.534 ± 0.252 | 0.583 |
| IL-13 | 9.393 ± 2.569 | 13.509 ± 1.494 | 6.878 ± 2.103 | 6.254 ± 1.620 | 0.198 |
| IFN-ϒ | 5.353 ± 0.848 | 5.804 ± 0.411 | 4.389 ± 1.482 | 1.976 ± 0.892 | 0.171 |
| TNF-α | 6.033 ± 1.441 | 6.473 ± 0.001 | 4.557 ± 1.684 | 2.997 ± 0.855 | 0.505 |
|  | **Pa Ev at PEx, Donor 1** | **Clinical Stability, Donor 1** | **Pa Ev at PEx, Donor 2** | **Clinical Stability, Donor 2** | **P-value** |
| IL-1β | 5.337±0.454 | 5.693 ± 1.319 | 4.889 ± 0.522 | 4.538 ± 0.412 | 0.991 |
| IL-2 | 10.913 ± 5.468 | 8.204 ± 1.775 | 8.564 ± 1.672 | 9.166 ± 0.493 | 0.325 |
| IL-4 | 0.594 ± 0.058 | 0.643 ± 0.118 | 0.661 ± 0.087 | 0.657 ± 0.071 | 0.447 |
| IL-6 | 34.812 ± 11.310 | 36.470 ± 20.143 | 21.436 ± 16.993 | 12.579 ± 3.189 | 0.481 |
| IL-8 | 19288.64 ± 4279.183 | 16753.84 ± 3206.120 | 11852.460 ± 2348.669 | 13754.090 ± 3263.858 | 0.797 |
| IL-10 | 125.428 ± 56.076 | 79.220 ± 50.122 | 88.590 ± 58.004 | 112.856 ± 10.748 | 0.528 |
| IL-12p70 | 2.060 ± 0.272 | 2.314 ± 0.409 | 2.464 ± 0.279 | 2.605 ± 0.400 | 0.107 |
| IL-13 | 14.707 ± 2.311 | 12.820 ± 1.822 | 14.270 ± 2.655 | 15.333 ± 1.414 | 0.594 |
| IFN-ϒ | 9.438 ± 0.386 | 10.568 ± 2.500 | 10.699 ± 1.531 | 9.838 ± 0.515 | 0.800 |
| TNF-α | 19.118 ± 22.843 | 8.880 ± 2.123 | 10.114 ± 2.917 | 11.206 ± 0.735 | 0.268 |
|  | **Pa Ev at PEx with ETI, Donor 1** | **Clinical Stability with ETI, Donor 1** | **Pa Ev at PEx with ETI, Donor 2** | **Clinical Stability with ETI, Donor 2** | **P-value** |
| IL-1β | 3.202 ± 0.494 | 3.392 ± 0.477 | 2.463 ± 0.611 | 2.321 ± 0.840 | 0.912 |
| IL-2 | 4.645 ± 0.789 | 5.210 ± 1.050 | 3.490 ± 1.222 | 3.451 ± 1.839 | 0.551 |
| IL-4 | 0.364 ± 0.059 | 0.431 ± 0.115 | 0.298 ± 0.083 | 0.309 ± 0.125 | 0.266 |
| IL-6 | 7.523 ± 1.193 | 7.916 ± 2.669 | 4.031 ± 0.871 | 4.133 ± 1.294 | 0.667 |
| IL-8 | 2285.845 ± 412.168 | 2788.080 ± 607.155 | 1403.75 ± 578.273 | 1738.507 ± 1792.557 | 0.234 |
| IL-10 | 2.438 ± 1.339 | 9.178 ± 10.968 | 2.689 ± 4.093 | 8.593 ± 20.921 | 0.123 |
| IL-12p70 | 1.155 ± 0.203 | 1.341 ± 0.406 | 0.950 ± 0.298 | 1.027 ± 0.559 | 0.331 |
| IL-13 | 9.393 ± 2.569 | 9.314 ± 1.898 | 6.878 ± 2.103 | 8.401 ± 2.887 | 0.393 |
| IFN-ϒ | 5.353 ± 0.848 | 5.971 ± 0.882 | 4.389 ± 1.482 | 4.739 ± 2.048 | 0.613 |
| TNF-α | 6.033 ± 1.441 | 6.654 ± 1.005 | 4.557 ± 1.684 | 5.257 ± 2.771 | 0.302 |
|  | **Pa Ev no ETI at PEx, Donor 1** | **Pa Ev with ETI at PEx, Donor 1** | **Pa Ev no ETI at PEx, Donor 2** | **Pa Ev with ETI at PEx, Donor 2** | **P-value** |
| IL-1β | 5.337 ± 0.468 | 3.202 ± 0.494 | 4.889 ± 0.522 | 2.463 ± 0.611 | <0.001* |
| IL-2 | 10.913 ± 5.892 | 4.635 ± 0.789 | 8.564 ± 1.672 | 3.490 ± 1.222 | <0.001* |
| IL-4 | 0.594 ± 0.055 | 0.364 ± 0.059 | 0.661 ± 0.087 | 0.298 ± 0.083 | <0.001* |
| IL-6 | 34.811 ± 11.309 | 7.523 ± 1.193 | 21.436 ± 16.993 | 4.031 ± 0.871 | <0.001* |
| IL-8 | 19288.64 ± 4279.183 | 2285.845 ± 412.168 | 11852.460 ± 2348.669 | 1403.75 ± 578.273 | <0.001* |
| IL-10 | 125.428 ± 51.445 | 2.438 ± 1.339 | 88.590 ± 58.004 | 2.689 ± 4.093 | <0.001* |
| IL-12p70 | 2.060 ± 0.272 | 1.155 ± 0.203 | 2.464 ± 0.279 | 0.950 ± 0.298 | <0.001* |
| IL-13 | 14.707 ± 2.311 | 9.393 ± 2.569 | 14.270 ± 2.655 | 6.878 ± 2.103 | <0.001* |
| IFN-ϒ | 9.438 ± 0.368 | 5.353 ± 0.848 | 10.699 ± 1.531 | 4.389 ± 1.482 | <0.001* |
| TNF-α | 19.118 ± 24.433 | 6.033 ± 1.441 | 10.114 ± 2.917 | 4.557 ± 1.684 | 0.022 |

GLS random-effects linear model with donor cells as the panel variable. * Adjusted p-value (Bonferroni correction) <0.00125. Pa, Pseudomonas aeruginosa; EV, extracellular vesicle; PEx, pulmonary exacerbation; MEM, minimal essential media; ETI, elexacaftor-tezacaftor-ivacaftor; IL, interleukin; IFN, interferon; TNF, tumor necrosis factor; NP, not performed.

**Supplemental Table 2. Measures of basal media inflammatory cytokines following *Pseudomonas aeruginosa* extracellular vesicle exposure**

| **Cytokine**  **(mean, SD)** | **Pa Ev at PEx, Donor 1** | **MEM control, Donor 1** | **Pa Ev at PEx, Donor 2** | **MEM control, Donor 2** | **P-value** |
| --- | --- | --- | --- | --- | --- |
| IL-1β | 5.369 ± 0.500 | 5.968 ± 0.283 | 5.371 ± 0.156 | NP | 0.024 |
| IL-2 | 11.950 ±1.602 | 13.564 ± 2.501 | 12.479 ± 0.992 | NP | 0.205 |
| IL-4 | 0.586 ± 0.060 | 0.683 ± 0.052 | 0.647 ± 0.083 | NP | 0.240 |
| IL-6 | 17.796 ± 4.615 | 36.316 ± 6.406 | 8.273 ± 1.542 | NP | <0.001* |
| IL-10 | 122.641 ± 34.477 | 159.650 ± 4.075 | 162.177 ± 17.352 |  | 0.765 |
| IL-12p70 | 2.195 ± 0.309 | 2.403 ± 0.370 | 2.221 ± 0.284 | NP | 0.376 |
| IL-13 | 19.379 ± 2.654 | 25.816 ± 0.633 | 18.326 ± 4.121 | NP | 0.005 |
| IFN-ϒ | 9.662 ± 0.540 | 10.437 ± 0.486 | 10.195 ± 0.461 | NP | 0.220 |
| TNF-α | 11.772 ± 1.378 | 13.883 ± 0.473 | 12.555 ± 2.367 | NP | 0.217 |
|  | **Pa Ev at PEx with ETI, Donor 1** | **MEM control with ETI, Donor 1** | **Pa Ev at PEx with ETI, Donor 2** | **MEM control with ETI, Donor 2** | **P-value** |
| IL-1β | 2.936 ± 0.374 | 3.991 ± 0.483 | 2.909 ± 0.479 | 2.548 ± 1.560 | 0.329 |
| IL-2 | 6.530 ± 0.621 | 9.335 ± 0.793 | 6.885 ± 0.863 | 6.099 ± 2.377 | 0.127 |
| IL-4 | 0.300 ± 0.065 | 0.459 ± 0.095 | 0.331 ± 0.066 | 0.267 ± 0.129 | 0.309 |
| IL-6 | 4.832 ± 0.855 | 6.988 ± 1.868 | 3.836 ± 0.654 | 3.663 ± 1.805 | 0.095 |
| IL-10 | 1.661 ± 0.418 | 12.409 ± 11.036 | 2.257 ± 0.795 | 2.541 ± 2.610 | 0.006 |
| IL-12p70 | 0.998 ± 0.275 | 1.692 ± 0.344 | 1.024 ± 0.161 | 0.886 ± 0.419 | 0.101 |
| IL-13 | 10.411 ± 2.925 | 17.989 ± 4.519 | 10.603 ± 2.305 | 9.731 ± 5.224 | 0.081 |
| IFN-ϒ | 4.792 ± 0.798 | 6.784 ± 1.349 | 5.373 ± 0.876 | 4.041 ± 2.232 | 0.620 |
| TNF-α | 4.886 ± 0.992 | 7.920 ± 2.223 | 5.004 ± 1.215 | 3.930 ± 2.660 | 0.269 |
|  | **Pa Ev at PEx, Donor 1** | **Clinical Stability, Donor 1** | **Pa Ev at PEx, Donor 2** | **Clinical Stability, Donor 2** | **P-value** |
| IL-1β | 5.369 ± 0.500 | 5.370 ± 0.332 | 5.371 ± 0.156 | 5.250 ± 0.329 | 0.619 |
| IL-2 | 11.950 ±1.602 | 12.079 ± 1.220 | 12.479 ± 0.992 | 12.601 ± 0.491 | 0.753 |
| IL-4 | 0.586 ± 0.060 | 0.599 ±0.089 | 0.647 ± 0.083 | 0.680 ± 0.109 | 0.436 |
| IL-6 | 17.796 ± 4.615 | 16.531 ± 4.860 | 8.273 ± 1.542 | 9.684 ± 3.247 | 0.957 |
| IL-10 | 122.641 ± 34.477 | 137.000 ± 19.316 | 162.177 ± 17.352 | 170.546 ± 15.314 | 0.154 |
| IL-12p70 | 2.195 ± 0.309 | 2.278 ± 0.271 | 2.221 ± 0.284 | 2.188 ± 0.358 | 0.814 |
| IL-13 | 19.379 ± 2.654 | 17.177 ± 3.252 | 18.326 ± 4.121 | 19.964 ± 3.339 | 0.817 |
| IFN-ϒ | 9.662 ± 0.540 | 9.779 ± 0.527 | 10.195 ± 0.461 | 10.233 ± 0.346 | 0.636 |
| TNF-α | 11.772 ± 1.378 | 12.172 ± 2.039 | 12.555 ± 2.367 | 13.076 ± 1.649 | 0.484 |
|  | **Pa Ev at PEx with ETI, Donor 1** | **Clinical Stability with ETI, Donor 1** | **Pa Ev at PEx with ETI, Donor 2** | **Clinical Stability with ETI, Donor 2** | **P-value** |
| IL-1β | 2.936 ± 0.374 | 3.247 ± 0.530 | 2.909 ± 0.479 | 3.288 ± 0.935 | 0.102 |
| IL-2 | 6.530 ± 0.621 | 7.301 ± 1.203 | 6.885 ± 0.863 | 7.931 ± 2.020 | 0.043 |
| IL-4 | 0.300 ± 0.065 | 0.338 ± 0.059 | 0.331 ± 0.066 | 0.393 ± 0.121 | 0.078 |
| IL-6 | 4.832 ± 0.855 | 5.262 ± 1.280 | 3.836 ± 0.654 | 4.254 ± 0.815 | 0.189 |
| IL-10 | 1.661 ± 0.418 | 2.977 ± 1.772 | 2.257 ± 0.795 | 28.689 ± 56.417 | 0.169 |
| IL-12p70 | 0.998 ± 0.275 | 1.117 ± 0.202 | 1.024 ± 0.161 | 1.205 ± 0.310 | 0.074 |
| IL-13 | 10.411 ± 2.925 | 10.548 ± 1.653 | 10.603 ± 2.305 | 12.533 ± 3.237 | 0.261 |
| IFN-ϒ | 4.792 ± 0.798 | 5.598 ± 0.856 | 5.373 ± 0.876 | 6.166 ± 1.817 | 0.048 |
| TNF-α | 4.886 ± 0.992 | 5.741 ± 1.186 | 5.004 ± 1.215 | 6.363 ± 2.247 | 0.032 |
|  | **Pa Ev no ETI at PEx, Donor 1** | **Pa Ev with ETI at PEx, Donor 1** | **Pa Ev no ETI at PEx, Donor 2** | **Pa Ev with ETI at PEx, Donor 2** | **P-value** |
| IL-1β | 5.369 ± 0.500 | 2.936 ± 0.374 | 5.371 ± 0.156 | 2.909 ± 0.479 | <0.001* |
| IL-2 | 11.950 ± 1.602 | 6.530 ± 0.621 | 12.479 ± 0.992 | 6.885 ± 0.863 | <0.001* |
| IL-4 | 0.586 ± 0.060 | 0.300 ± 0.065 | 0.647 ± 0.083 | 0.331 ± 0.066 | <0.001* |
| IL-6 | 17.796 ± 4.615 | 4.832 ± 0.855 | 8.273 ± 1.542 | 3.836 ± 0.654 | <0.001* |
| IL-10 | 122.641 ± 34.477 | 1.661 ± 0.418 | 162.177 ± 17.352 | 2.257 ± 0.795 | <0.001* |
| IL-12p70 | 2.195 ± 0.309 | 0.998 ± 0.275 | 2.221 ± 0.284 | 1.024 ± 0.161 | <0.001* |
| IL-13 | 19.379 ± 2.654 | 10.411 ± 2.925 | 18.326 ± 4.121 | 10.603 ± 2.305 | <0.001* |
| IFN-ϒ | 9.662 ± 0.540 | 4.792 ± 0.798 | 10.195 ± 0.461 | 5.373 ± 0.876 | <0.001* |
| TNF-α | 11.772 ± 1.378 | 4.886 ± 0.992 | 12.555 ± 2.367 | 5.004 ± 1.215 | <0.001* |

GLS random-effects linear model with donor cells as the panel variable. *Adjusted p-value (Bonferroni correction) <0.00125. Pa, Pseudomonas aeruginosa; EV, extracellular vesicle; PEx, pulmonary exacerbation; MEM, minimal essential media; ETI, elexacaftor-tezacaftor-ivacaftor; IL, interleukin; IFN, interferon; TNF, tumor necrosis factor; NP, not performed. IL-8 results were above the upper limit of quantification for the assay (data not shown).


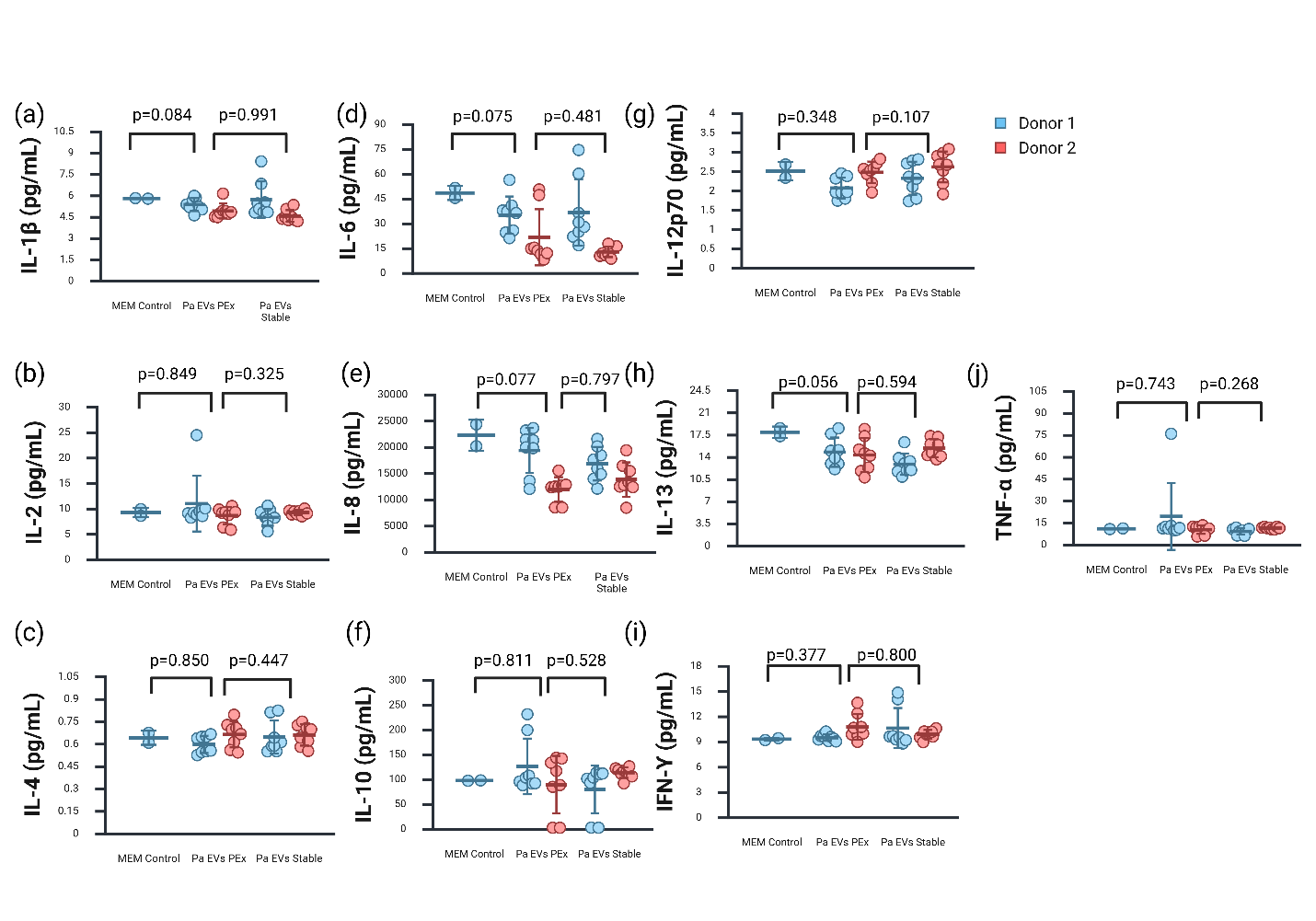


**Supplemental Figure 1. Inflammatory cytokines in the apical secretions of primary cystic fibrosis cells exposed to controls and *Pseudomonas aeruginosa* extracellular vesicles isolated from times of clinical stability and pulmonary exacerbation in the absence of elexacaftor-tezacaftor-ivacaftor (ETI) treatment.** Error bars represent the standard deviation surrounding the mean. P-values shown are for the GLS random-effects linear model for the dependent variable ETI exposure, and setting donor as the panel variable. (a) Interleukin (IL)-1β. (b) IL-2. (c) IL-4. (d) IL-6. (e) IL-8. (f) IL-10. (g) IL-12p70. (h) IL-13. (i) Interferon (IFN)-Ɣ. (j) Tumor necrosis factor (TNF)-α. Created in BioRender. Hahn, A. (2026) https://BioRender.com/7fpqhz5


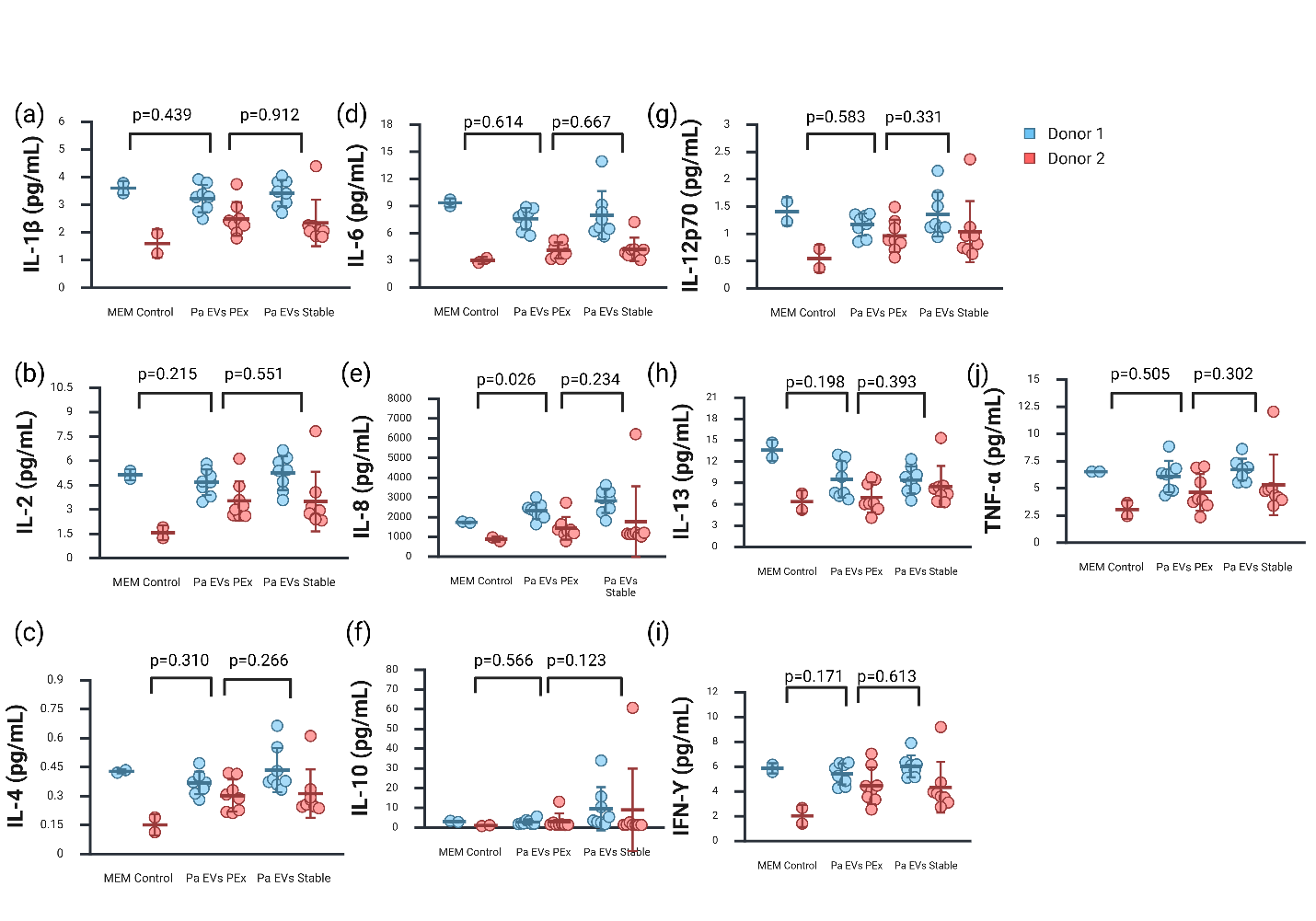


**Supplemental Figure 2. Inflammatory cytokines in the apical secretions of primary cystic fibrosis cells exposed to controls and *Pseudomonas aeruginosa* extracellular vesicles isolated from times of clinical stability and pulmonary exacerbation in the presence of elexacaftor-tezacaftor-ivacaftor (ETI) treatment.** Error bars represent the standard deviation surrounding the mean. P-values shown are for the GLS random-effects linear model for the dependent variable ETI exposure, and setting donor as the panel variable. (a) Interleukin (IL)-1β. (b) IL-2. (c) IL-4. (d) IL-6. (e) IL-10. (f) IL-12p70. (g) IL-13. (h) Interferon (IFN)-Ɣ. (i) Tumor necrosis factor (TNF)-α. IL-8 results were above the upper limit of quantification for the assay (data not shown). Created in BioRender. Hahn, A. (2026) https://BioRender.com/ooyuaaj


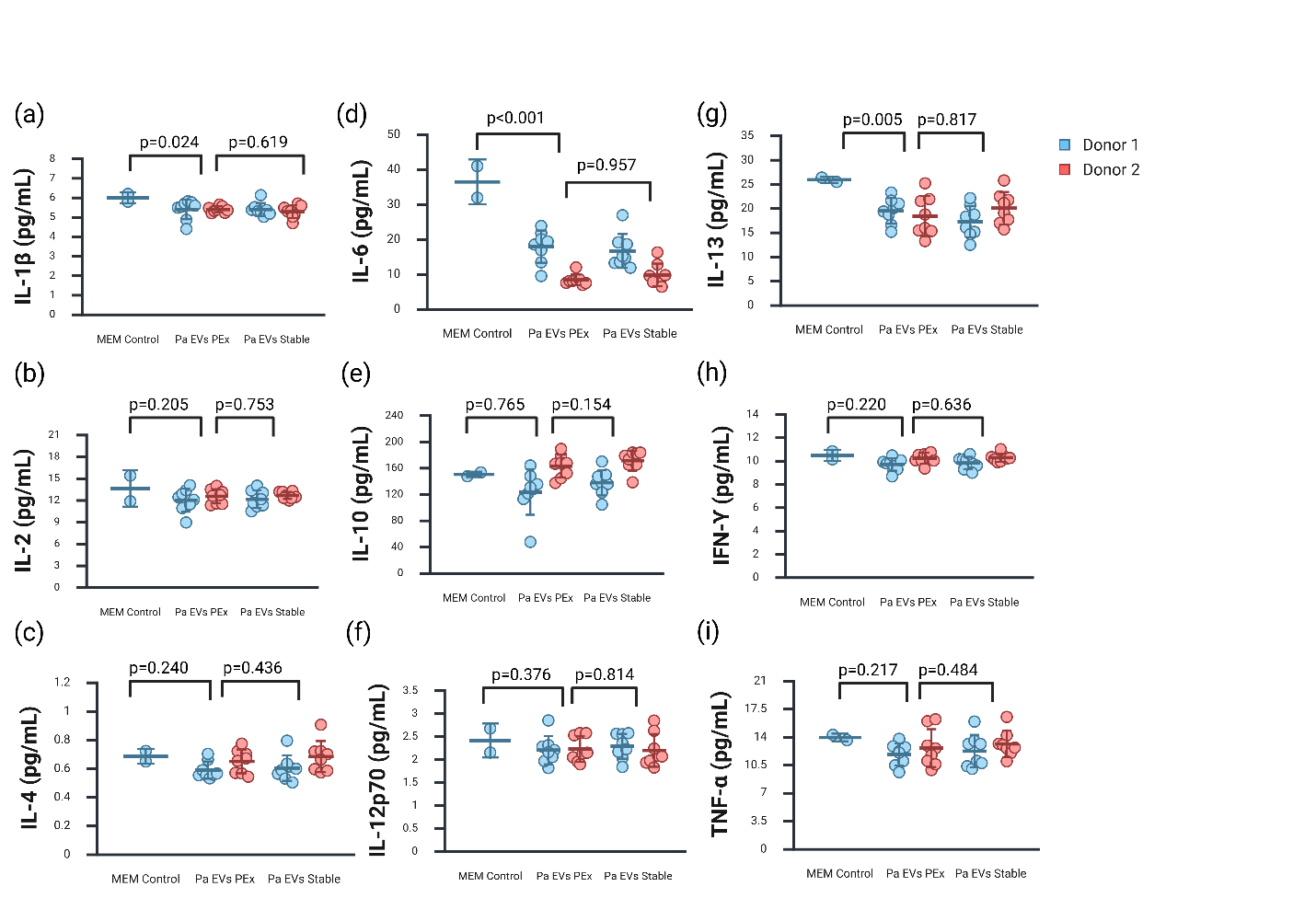


**Supplemental Figure 3. Inflammatory cytokines in the basal media of primary cystic fibrosis cells exposed to controls and *Pseudomonas aeruginosa* extracellular vesicles isolated from times of clinical stability and pulmonary exacerbation in the absence of elexacaftor-tezacaftor-ivacaftor (ETI) treatment.** Error bars represent the standard deviation surrounding the mean. P-values shown are for the GLS random-effects linear model for the dependent variable ETI exposure, and setting donor as the panel variable. (a) Interleukin (IL)-1β. (b) IL-2. (c) IL-4. (d) IL-6. (e) IL-8. (f) IL-10. (g) IL-12p70. (h) IL-13. (i) Interferon (IFN)-Ɣ. (j) Tumor necrosis factor (TNF)-α. Created in BioRender. Hahn, A. (2026) https://BioRender.com/68z69l9


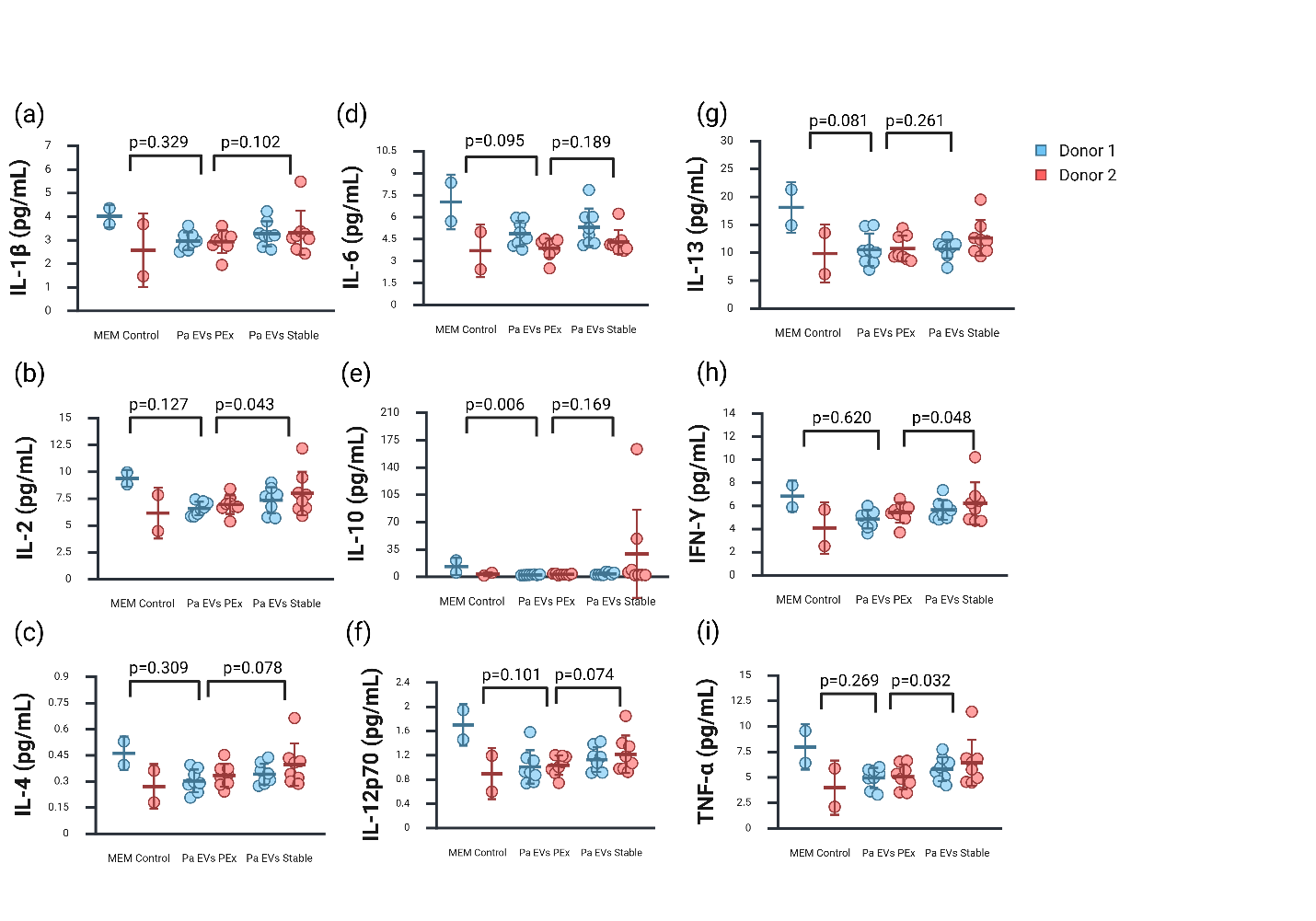


**Supplemental Figure 4. Inflammatory cytokines in the basal media of primary cystic fibrosis cells exposed to controls and *Pseudomonas aeruginosa* extracellular vesicles isolated from times of clinical stability and pulmonary exacerbation in the presence of elexacaftor-tezacaftor-ivacaftor (ETI) treatment.** Error bars represent the standard deviation surrounding the mean. P-values shown are for the GLS random-effects linear model for the dependent variable ETI exposure, and setting donor as the panel variable. (a) Interleukin (IL)-1β. (b) IL-2. (c) IL-4. (d) IL-6. (e) IL-10. (f) IL-12p70. (g) IL-13. (h) Interferon (IFN)-Ɣ. (i) Tumor necrosis factor (TNF)-α. IL-8 results were above the upper limit of quantification for the assay (data not shown). Created in BioRender. Hahn, A. (2026) https://BioRender.com/w1oia0n


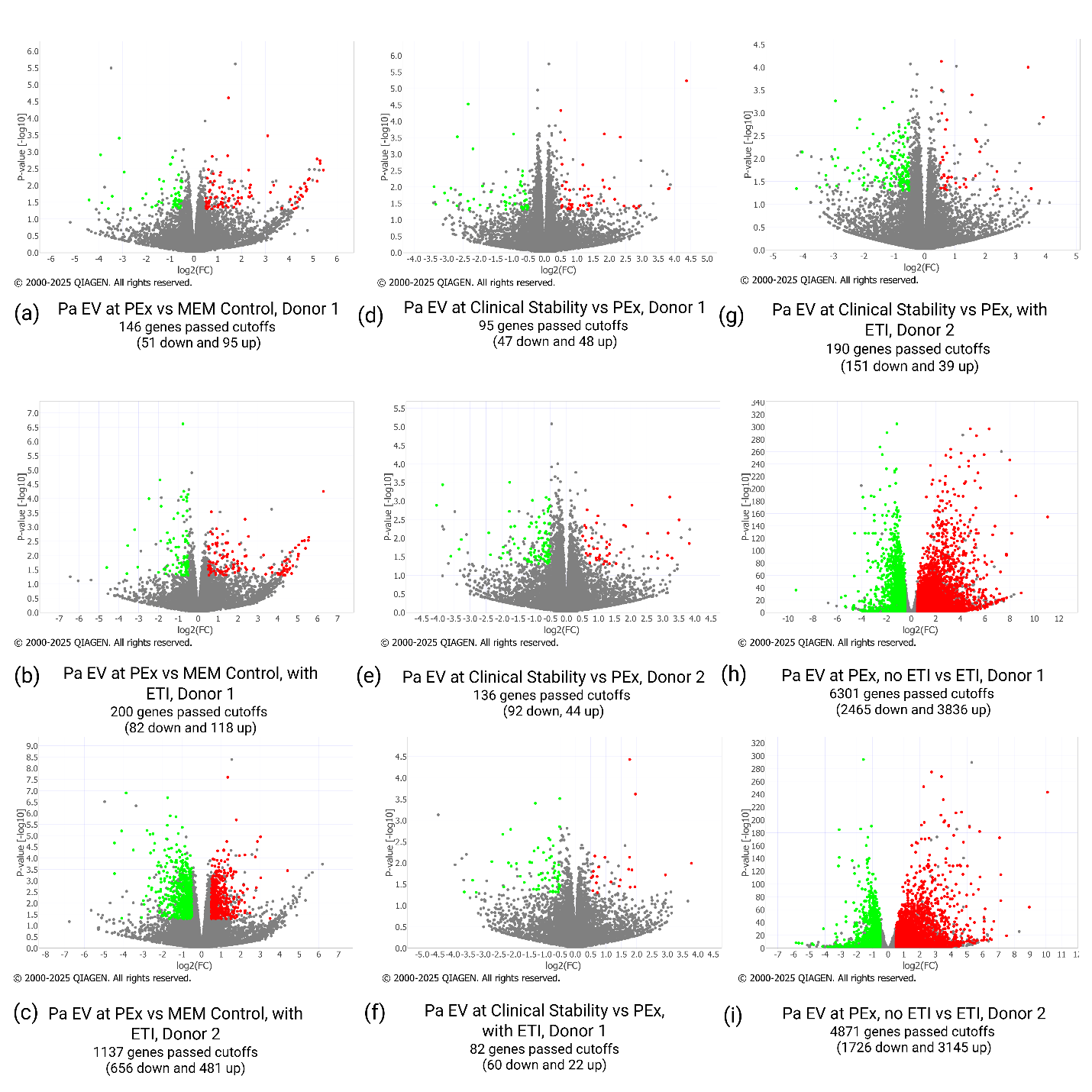


**Supplemental Figure 5. Differentially abundant genes incorporated into Ingenuity Pathway Analysis.** (a) Pa EV at PEx vs MEM control, Donor 1. (b) Pa EV at PEx vs MEM control, with ETI, Donor 1. (c) Pa EV at PEx vs MEM control, with ETI, Donor 2. (d) Pa EV at Clinical Stability vs PEx, Donor 1. (e) Pa EV at Clinical Stability vs PEx, Donor 2. (f) Pa EV at Clinical Stability vs PEx, with ETI, Donor 1. (g) Pa EV at Clinical Stability vs PEx, with ETI, Donor 2. (h) Pa EV at PEx, no ETI vs with ETI, Donor 1. (i) Pa EV at PEx, no ETI vs with ETI, Donor 2. Genes that were up-regulated (red) are higher in the first comparator, whereas genes that were down-regulated (blue) are higher in the second comparator. Pa, Pseudomonas aeruginosa; EV, extracellular vesicles; PEx, pulmonary exacerbation; MEM, minimal essential media; ETI, elexacaftor-tezacaftor-ivacaftor. Figure generated in Ingenuity Pathway Analysis (Qiagen) and created in BioRender. Hahn, A. (2026) https://BioRender.com/rfk9k25
